# Perspectives of type 2 diabetes mellitus management in Algeria: a comprehensive expert review

**DOI:** 10.3389/fcdhc.2025.1495849

**Published:** 2025-04-15

**Authors:** Mohamed Belhadj, Rachid Malek, Houssem Baghous, Mourad Boukheloua, Zakia Arbouche, Nassim Nouri, Mohammed El Amine Amani, Fethia Sersoub, Mohamed Amine Haireche

**Affiliations:** ^1^ Internal Medicine Department, Specialist Hospital 1^er^ Novembre 1954, Oran, Algeria; ^2^ Internal Medicine Department, University of Medical Sciences Ferhat Abbas, Setif, Algeria; ^3^ Department of Diabetology, Mustapha Bacha Hospital, Algiers, Algeria; ^4^ Department of Cardiology, Nafissa Hamoud Hospital (ex Parnet), Algiers, Algeria; ^5^ Private Practitioner, Algiers, Algeria; ^6^ Diabetology Department, Medicine Faculty of Constantine, Constantine, Algeria; ^7^ Medical Department, Hikma Pharma, Algiers, Algeria; ^8^ Medical Writing Department, Medical Axès, Boulogne Billancourt, France

**Keywords:** type 2 diabetes mellitus, Algeria, burden, socioeconomic factors, prevention, management strategy, epidemiology

## Abstract

**Background:**

The health and economic impacts of type 2 diabetes mellitus (T2DM) remain substantial, notably in developing countries.

**Objectives:**

To provide an in-depth assessment of the T2DM situation in Algeria to understand its multifaceted burden and identify priority areas of intervention.

**Methods:**

A systematic literature search was conducted on all published articles about T2DM in Algeria over the past 30 years, including original research, reviews, and case series. The extracted data were thoroughly analyzed and synthesized by a committee of diabetes experts.

**Results:**

Algerian epidemiological data point towards a constant rise of T2DM prevalence, roughly from 8.9% in 2003 to 14.4% in 2016-2017. The mean onset is around 41 years with women experiencing a greater burden at younger age. Low socioeconomic status, limited education, and lack of health insurance exacerbate T2DM risk and health inequities. Lifestyle and metabolic risk factors are prevalent. Despite advancements in glycemic control, prescribing practices lack standardization, with suboptimal use of antidiabetic drugs and absence of novel drugs in the market. Health and economic burdens are dominated by complications, highlighting inadequate primary and tertiary prevention strategies.

**Conclusion:**

Notwithstanding the increasing burden of T2DM in Algeria, the healthcare strategies and therapeutic outcomes remain suboptimal. This underscores the necessity for a comprehensive strategy including enhanced prevention, access to novel treatments, standardized practices, along with a patient-centered approach.

## 1 Introduction

Type 2 diabetes mellitus (T2DM) is one of the most prevalent chronic disorders of adulthood worldwide (1). Its recent (2021) global prevalence was estimated at 10.5% (536.6 million people) in the 20-79 age group, projected to increase to 12.2% (783.2 million) by 2045 (2). Other data estimated the global prevalence at 437.9 million cases in 2019, resulting in 1.5 million deaths and 66.3 million disability-adjusted life years (DALYs); however, T2DM burden is highest in low- and middle-income countries such as Algeria (3).

Another aspect of diabetes burden is the elevated cardiovascular risk. Despites advancements that reduced the burden of microvascular complications, cardiovascular diseases persist as the predominant cause of death in T2DM patients (4). It is imperative to highlight that T2DM exacerbates the progression of atherosclerotic plaque formation and its subsequent rupture, a critical precipitating factor for acute cardiovascular incidents such as myocardial infarction and cerebrovascular strokes (5, 6).

T2DM pathophysiology involves complex interactions leading to β-cell dysfunction, insulin resistance, and hyperglycemia. Key features include diminished insulin secretion and β-cell insensitivity to glucose, alongside increased glucagon levels and disrupted incretin hormones, contributing to poor glycemic control (7–10). Recent evidence highlights the major role of obesity in inducing insulin resistance in peripheral tissues, by disrupting insulin signaling subsequent to high free fatty acids levels and adipokines secretion (11, 12). This generates a vicious cycle where insulin resistance increases gluconeogenesis and lipolysis, leading to hyperglycemia and elevated free fatty acids. In chronic stages, T2DM induces oxidative stress and endoplasmic reticulum stress in β-cells, resulting in their apoptosis (10, 13, 14). Additionally, systemic inflammation, altered lipid metabolism, and neurotransmitter dysfunction due to central insulin resistance are significant in T2DM progression (7, 15).

These new insights enabled considerable advancements in diabetes pharmacopeia and management approaches. The emergence of novel hypoglycemic drugs, such as GLP-1 receptor agonists (GLP-1RA), DPP-4 inhibitors (DPP-4i), and SGLT-2 inhibitors (SGLT-2i), has not only improved glycemic control but also provided benefits in terms of cardiovascular and renal protection (16–18). The utilization of these novel drugs is increasingly integrated in clinical practice guidelines, along with particular emphasis on weight reduction and dietary and other lifestyle changes (19).

Algeria exemplifies developing countries facing escalating challenges from T2DM and its associated complications. Despite significant local efforts, the situation is alarming due to rising disease prevalence, high obesity and overweight rates, inadequate glycemic control, and substantial undiagnosed cases (20–23). The estimated T2DM prevalence in Algeria is 10% based on previous regional studies, but updated data is needed to account for a probable significant increase in cases in recent years. Predictably, this prevalence is higher among old individuals and those living in urban areas and those with positive familial history (20, 24). Besides, the national economic cost of T2DM is high, and expenditures are essentially dedicated to the management of complications and medication supply (25). It is therefore crucial to identify the effective and cost-effective strategies to optimize the prevention and management strategies of T2DM in Algeria.

The present review aims to propose solutions for enhancing the management of T2DM in Algeria. It provides a comprehensive analysis of T2DM burden and management indicators in Algeria, thereby characterizing the current and future challenges and barriers that impede the improvement in patients with diabetes care. Such analysis holds significant importance in strategic planning and resource allocation in both preventive and therapeutic programs.

## 2 Methodology

A systematic literature search was conducted to include published data addressing T2DM epidemiology, risk factors, management, and outcomes in Algeria over the past three decades. Published articles were searched in PubMed/MEDLINE, Embase, Scopus, Web of Science, and Google Scholar. We used a comprehensive literature search strategy employing keywords used “Algeria”, “Algerian”, “Type 2 Diabetes”, along with Boolean operators (AND, OR). Referenced conference posters and academic dissertations providing original and relevant data were also included, sourced from a committee of diabetes experts.

We included all original or review articles or case studies in English or French, based on observational or interventional data, conducted in Algeria or involving Algerian patients as part of a multinational project. However, case reports were excluded.

Two teams of two independent reviewers conducted the literature screening. The first team independently screened the titles and abstracts of the identified articles to determine their relevance based on the inclusion criteria. The second team retrieved full-text articles of potentially relevant studies and assessed them for final inclusion in the review. The studies were classified by chapter, as per relevance; knowing that one study may be relevant to more than one chapter. Data extraction was conducted by a team of three investigators. Key data were synthesized using a narrative and descriptive synthesis approach combined with a thematic analysis, to identify trends, patterns, and figures. Where relevant, findings were structured chronologically and categorized by themes. Results are organized into four chapters and a general discussion.

## 3 Epidemiological figures of T2DM in Algeria

**Figure 1:**
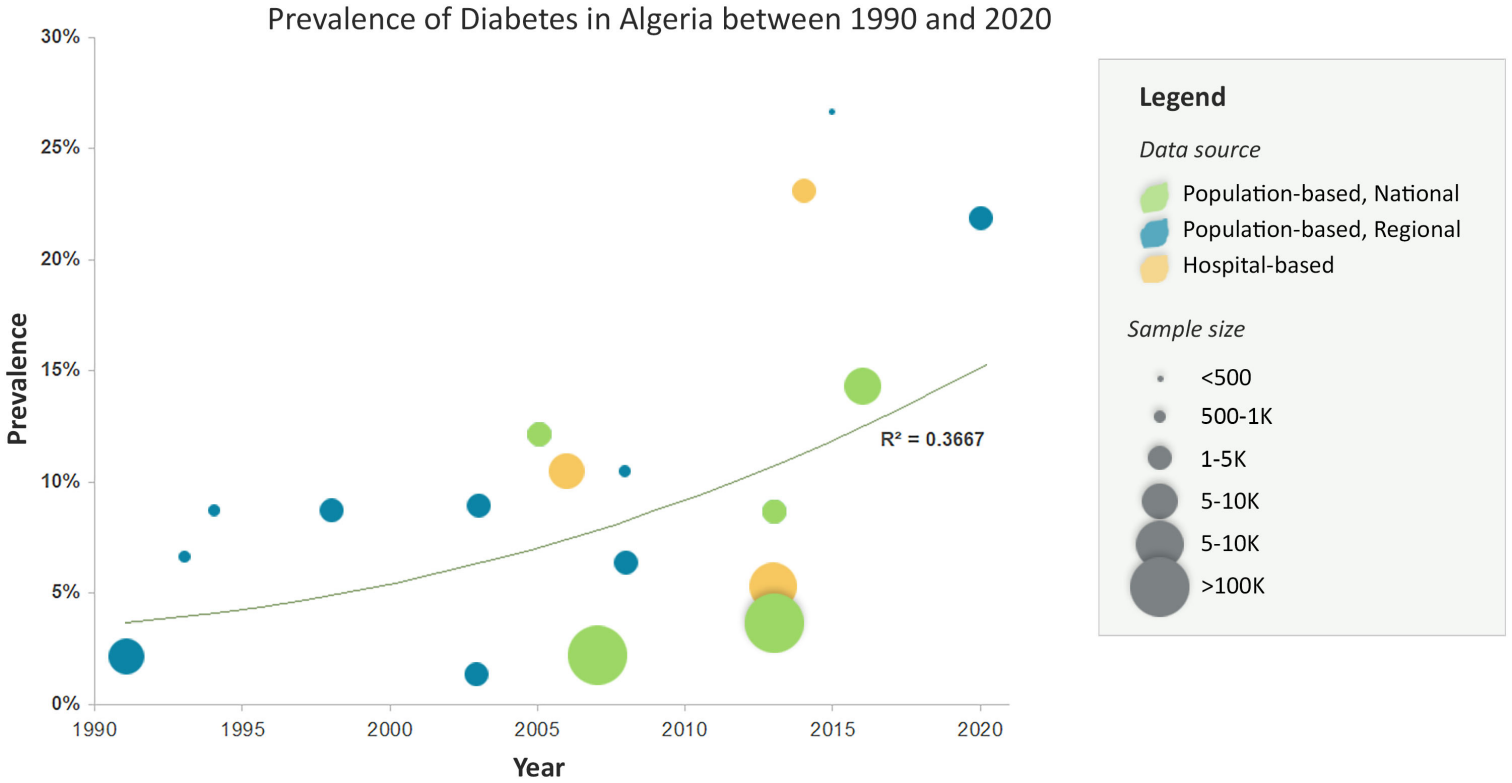
Prevalence of type 2 diabetes in Algeria between 1990-2020 using various estimates and data sources.

During the past three decades, the prevalence of T2DM in Algeria has been examined through various population-based and hospital-based studies. However, the estimates and their reliability fluctuated depending on the methodology used, noting a big heterogeneity in the studied populations and diagnostic methods. Malek reported the most important studies published up to 2005 (26). In this section, we will review the available data on T2DM prevalence in Algeria, considering the different study designs and their implications. The different prevalence estimates and trends are summarized in **Figure 1**, by specifying the recruitment method (population- versus hospital-based, regional versus national) and sample size of the different studies.

### 3.1 National, population-based studies

T2DM prevalence in Algeria has been rising over the past three decades, as demonstrated by the WHO StepWise project and other large-scale national studies. In 2003, the StepWise WHO-Algeria project was piloted in two regions, Mostaganem and Setif, involving 4,050 individuals aged 25-64 years. The pilot survey reported a T2DM prevalence of 8.9% based on elevated fasting blood glucose (27). In 2005, the National Institute of Public Health conducted the Tahina project (published in 2007), a national survey involving 4,818 individuals aged 35-70 from 26 districts. The survey revealed a higher prevalence of 12.20% based on the same criteria. Another remarkable finding was the early onset of T2DM from the age of 30, with no difference in prevalence between the two genders, along with a lower prevalence in the south of Algeria (28).

In 2006, another large-scale, household national study, the Multiple Indicator Cluster Survey 3 (MICS 3) was conducted by the Algerian Ministry of Health (MOH), in collaboration with the United Nations Children’s Fund (UNICEF) and the United Nations Population Fund (UNFPA). MICS 3 surveyed 29,008 households and found a prevalence of 2.1% (29). In the same year, Malek et al. conducted a large-scale (N=86,785) screening campaign (Published in 2013), involving 86,785 participants aged 35 years old or older, in Eastern and Southeastern Algeria. The estimated prevalence of unknown T2DM was 5.27% (30), which should be added to the overall prevalence.

In 2013, MICS 4 survey was conducted involving 147,471 individuals, including 98,539 adults. The overall prevalence of T2DM was 2.9% while that among individuals aged 25 and older was 3.6%, gradually increasing to reach 10.7% in age category 60-69 years (31). Conversely, Handlos et al. (32) conducted an international population-based study involving 2155 Algerian participants, among 6588 total participants, which reported a prevalence of 8.70% based on HbA1c levels (32). In 2016-2017, a second edition of the StepWise WHO-Algeria survey was conducted among 7,450 individuals aged 25-64 years from various regions in Algeria. The survey indicated a prevalence of 14.40% based on elevated FBG levels, representing a 1.6-fold increase by reference to 2003 (approximately 10 years later) (33).

### 3.2 Regional, population-based studies

Besides these national studies, several regional, population-based studies have been conducted in the past 3 decades, involving various methods and subgroups. These studies showed that T2DM prevalence has been on the rise since the late 1990s and fluctuating around 8% in the early 2000s. Notably, two studies that used oral glucose tolerance test (OGTT) supported these findings: one by Houti et al. in Oran in 1994 (34), and another by Malek et al. in Setif in 1998 (35), reporting a prevalence of 6.3% and 8.8%, respectively. Consistently, Kamali Z et al. reported a prevalence of 8.70% among individuals above the age of 25 in the district of Beb El Oued district, Algiers, in 1994 (36). These figures should be added those of undiagnosed T2DM, reported to be 2.10% in Algiers, in 1992 (37). Nonetheless, we note some significant regional variations in T2DM prevalence during that period. For instance, in 2003, Belhadj M et al. observed a remarkably lower prevalence of 1.30% among the Touareg population in Adrar, using FBG levels (38).

Studies that used other diagnostic methods of T2DM showed variable figures. In 2008, Yahia-Berrouiguet et al. reported a prevalence of 6.40% in Tlemcen, among individuals aged 25 years and above, based on elevated FBG levels or oral antidiabetic medication use (39). In 2015, Chami et al. reported a higher prevalence of 26.7% in Sidi Bel Abbas, among 393 individuals aged ≥65 years using fasting and random capillary glucose levels (40). The latter figure should consider the study focus on older population and the limits of the used diagnostic methods. However, it suggests the high prevalence of undiagnosed cases among this age category. More recently, Dalichaouche-Benchaoui et al. conducted a study on the metabolic and systemic cardiovascular risk factors associated with obesity among 1,200 adult inhabitants of Constantine, in Eastern Algeria. Among the outcomes was the prevalence of diabetes, which was estimated to be as high as 21.91% (41).

### 3.3 Hospital-based studies

In the meantime, several hospital-based studies have explored the prevalence of T2DM, targeting various populations. Zaoui S et al. (24) conducted a multicenter study in Tlemcen, including 7,656 individuals above 20 years old. They reported a prevalence of 10.50% based on elevated FBG levels (24). In 2014, Azzouz et al. conducted a hospital-based study in Algiers, reporting a prevalence of 23.10% among individuals aged 35 years and older using FBG levels and/or glucose tolerance tests (42). Additionally, Chami et al. (40) conducted a population-based study in Sidi Bel Abbas, which revealed a high prevalence of 26.7% among individuals aged 65 years and older based on capillary blood glucose levels (40).

A few other hospital-based studies with a less rigorous designs have been conducted. In 2017, Bendib et al. recruited 200 hypertensive patients in a single-center study, and found a frequency of T2DM as high as 46.50% (35). Finally, Nebab et al. conducted a multicenter study involving 3,547 adult individuals (18 years and older) who were attending hospital clinics for routine visits, and found that 47.3% were known diabetics (43). Although the two latter studies are not reliable in reflecting the prevalence, they are indicative of the substantial T2DM comorbidity and associated expenditures in terms of financial, logistic and human resources incurred by the health system.

## 4 Socioeconomic determinants and risk factors of T2DM in Algeria

A total of 28 studies provided data on T2DM risk factors in Algeria, between 2007 and 2022. The main findings are presented in **Table 1** and synthesized in the present section to enable defining high-risk groups.

**Table 1:** Systematic review of risk factors associated with type two diabetes in Algeria between 2004 and 2022.

Reference (Authors)	Year	Place	Design	Population characteristics	Risk factors^§^
Benotmane et al. (44)	2004	Oran [West]	Hospital-based	339 hospitalized diabetic patients with foot lesions	86.8% of men with diabetic foot lesion were smokers
Zaoui et al. (24)	2007	Tlemcen [West]	Hospital-based (MS)	7,656 aged > 20 years	Male gender, urban residents, positive family history, obesity
Ministry of Health (29)	2008	National (**MICS 3**)	Population-based	~171,150 participants	Older age, urban setting, high economic class, Relatively lower prevalence risk in Southern regions (1.8%) versus center (2.3%)
Houti et al. (45)	2009	Oran [West]	Population-based	787 aged 30-64	Older age, high BMI, Waist circumference, waist-to-hip ratio, high blood pressure measures
Belhadj et al. (46)	2010	National (**DiabCare**)	Hospital-based (MS)	1,005 diabetic patients following up for at least 12 months	Mean ± age of onset: 41.25 ± 13.69 years 74.1% were overweight or obese Cardiovascular risk factors: hypertension 32.5% (6.0% non-treated); low HDL 54,2%, hypertriglyceridemia 32.2%; high LDL 95.2%; Smoking 9.3%
Belmokhtar et al. (47)	2011	Maghnia, Tlemcen [West]	Hospital based, case-control	551 diabetic patients and 271 controls	Low educational level, low economic level (income <100.000DZD), number of children >5, high number of people in household, obesity, sedentary lifestyle, irregular food intake, hypertension, and family history of diabetes, **no health insurance**
Sahi et al. (48)	2011	Maghnia, Tlemcen [West]	Hospital-based	280 diabetics and 271 controls	No association with ABO/Rh blood group
Handlos et al. (32)	2013	International (Algeria, UAE and Saudi Arabia)	Population-based	6,588 individuals (2155 Algerians) aged between 30 and 75	Older age, high BMI. Algeria had the lowest rate of current smokers (12.5%).
Malek et al. (30)	2013	Eastern and south-eastern region (18 wilayas)	Hospital-based (MS)	86,785 individuals aged 35 and older, unknown diabetic status	Older age, family history of diabetes, history of gestational diabetes, hypertension, obesity, no influence of sex
Mohammed Nadjib et al. (49)	2013	Tlemcen [West]	Hospital-Based, case-control	150 individuals (90 diabetics and 10 controls)	Most affected age group is: 45–55 years (24.44%) in females and over 65 years (20%) in males, overweight and obesity, HbA1c increases with age in diabetics.
Azouz et al. (42)	2014	Alger [Center]	Hospital-based (MS)	1000 individuals aged 35 years or older	Male sex
Ouhaibi-Djellouli et al. (50)	2014	Oran [West]	Population-based	787 individuals aged between 30-64	*Gene effect*: T allele of the rs7903146 single nucleotide polymorphism (OR=1.55); *gene-diet interaction*: T allele carriers with high dessert and milk intakes (OR = 2.61).
Sebbani et al. (51)	2014	Tlemcen [West]	Hospital-based	73 individuals with depression	High prevalence of T2DM (69.9%; 95%CI: 57.9–79.8) in depression.
Diaf et al. (52)	2014	Sidi Bel Abbas and Mascara [West]	Hospital-based (MS)	238 diabetics aged 19 – 75, on oral antidiabetics	High proportion of females; positive correlation of high BMI with postprandial glucose, HGL-c and apolipoprotein A-I, and triglyceride levels.
Ministry of Health, UNICEF, UNFPA (31)	2015	National (**MICS 4**)	Population-based	147,471 participants, including 98539 adults	Older age: 40-49 (3.4%), 50-59 (8.1%), 60-69 (10.7%), 70+ (9.7%); no gender difference; no significant difference between urban and rural residents
Diaf et al. (53)	2015	Sidi Bel Abbas and Mascara [West]	Hospital-based (MS)	285 patients aged 20 – 75	Overweight and obesity more frequent in diabetic females than males, overweight and obese diabetics have higher total energy intake and lower meal frequency, and higher consumption of total fat, and saturated and polyunsaturated fatty acids.
Ferdi et al. (54)	2016	Tebessa [East]	Hospital-based, case-control (MS)	100 diabetics and 100 controls	Lower education, lower socioeconomic status, positive family history, no sex difference
Ferdi et al. (55)	2018	Tebessa [East]	Hospital-based, case-control (MS)	100 diabetics and 100 control	Older age (**not reliable**); higher BMI associated with higher blood glucose levels in diabetics.
Belhadj et al. (21)	2013-2017	National (Barometer)	Hospital-based	14,609 adult T2DM patients aged 18–97 years	Male-to-female ratio = 0.61; overweight (41.2%) and obesity (37.2%); hypertension (37.6%); dyslipidemia (28.2%); family history of diabetes (63%), CVD (36.1%),
Kachekouche et al. (56)	2018	Western region	Hospital based, case-control	1,059 diabetics and 793 controls aged 30 years or older	High mean corpuscular hemoglobin (>36 g/dL), lower platelets blood ratio, basophils ratio and sedimentation rate at one hour
Behar et al. (57)	2020	Tlemcen [West]	Hospital based, case-control	140 female diabetics and 150 female healthy controls	High (OR=2.21) and low (OR=2.52) dietary selenium intake
Bounihi et al. (58)	2021	Algiers [Center]	Hospital-based	390 diabetics	High frequency of obesity (41.3%) and abdominal obesity (93.5%), female sex, hypertension, low physical activity, high meat and protein intake, and higher dietary diversity score (DDS, OR for diabetes =1.38)
Mansouri et al. (59)	2021	Algiers [Center]	Hospital-based	180 diabetics and 181 controls aged 18-86	Chlorinated persistent organic pollutants: p,p’-dichlorodiphenyldichloroethylene (p,p’-DDE, OR: 12.58), hexachlorobenzene (HCB, OR: 3.69), polychlorinated biphenyl (PCB, OR: 2.28).
Dalichaouche-Benchaoui and Abadi (41)	2022	Constantine [East]	Population-based	1,200 aged 18 and older	Older age, higher BMI, triangular relationship with dyslipidemia
Nebab et al. (43)	2022	Multiregional	Hospital-based (MS)	3547 aged 18 and older	Obesity
Khaldi et al. (60)	2022	Ouargla [South]	Hospital-based (MS)	76 patients giving 103 clinical samples	The prevalence of ESBL and carbapenemase-producing *Enterobacteriaceae* was higher among diabetics (11.42% versus 2.85%) compared to non- diabetics respectively.
Hafidh et al. (61)	2022	International (12 countries)	Hospital-based (MS)	3,525 diabetics	Compared to MENA countries, Algerian diabetics have the lowest % of affected males (48.5%), lowest mean HbA1c (8.3%), lowest mean BMI (29.1 Kg/m^2^), lowest rate of current smokers (6.6%) and hyperlipidemia (19.9%). Microvascular complications (23.7%) - peripheral neuropathy was the most common, macrovascular complications (6.2%).
Mimoune et al. (62)	2022	Constantine [East]	Hospital-based	47 diabetics	High smoking rate (34%)

**^§^** Except otherwise specified, only categories with higher risk are mentioned.

(MS), Multicenter study; ‘diabetics’ refers to known type 2 diabetes; ESBL, Extended-Spectrum Beta-Lactamase producing bacteria.

### 4.1 Age and gender

According to the Barometer-Algeria study (N=14,609), conducted between 2013-2017 by Belhadj et al., the mean age of T2DM patients was 60.3 (SD=10.6) years (21). However, the age of onset, as reported in DiabCare study (N=1,005), is 41.25 (13.69) years (46). Older age is consistently identified as a risk factor for T2DM in the Algerian population (29–32, 41, 45, 49, 55). As to gender, we observed a mixed effect. While large screening and case-control studies observed no gender difference in the prevalence of T2DM (30, 31, 54), other studies reported either male (24, 42) or female (21, 53, 58) predominance. Nevertheless, compared to other Middle-East and North Africa (MENA) countries, the Algerian population with diabetes had the lowest male ratio (0.94) (61). We also observed an age-gender interaction, with females incurring a higher burden at a younger age (49). These data are highly valuable in designing targeted strategies.

### 4.2 Other social determinants

A few studies explored the other social determinants of diabetes in Algeria. Data before 2010 showed a higher risk of T2DM among urban residents (24, 29). Subsequent data show that rural residents experience equivalent burden, denoting a change in the lifestyle (31). Consistently, while T2DM used to be more frequent among high economic class groups (29), more recent studies showed a higher burden associated with low economic status, in addition to lower education and higher number of children (47, 54). These indicators suggest a change in T2DM epidemiology towards most disadvantaged groups, especially given their lower access to healthcare. In the study by Belmokhtar et al., not having a health insurance registration was found to be a significant risk factor for diabetes (47).

### 4.3 Nutritional and lifestyle factors

In the absence of longitudinal or population-based studies, several hospital-based studies have explored the association of T2DM with nutritional and lifestyle factors. The risk of T2DM is associated with sedentary lifestyle and poor dietary habits, including irregular, high-calory meals, lower meal frequency, and higher fat consumption; all being associated with increased body mass index (47, 53, 58). This is consistent with suboptimal dietary habits and physical activity among the Algerian population, as reported in the 2016-2017 StepWise study (33). Another case-control study by Behar et al. showed an association of T2DM with both high and low dietary selenium (57). Smoking rates varied largely across studies (46, 62); however, Algerian patients with diabetes showed to have the lowest rates of active smoking (6.6%), compared to other MENA countries (61).

### 4.4 Systemic conditions and metabolic risk factors

Up to 74.1% of Algerian patients with diabetes were found to be overweight or obese, while abdominal obesity was found in 93.5% (21, 46, 58). Several other studies reported high BMI, waist circumference and waist-to-hip ratio as risk factors for T2DM (24, 32, 47, 49). These figures are relatively higher than those in the general population, where overweight and obesity were found in approximately 56% of the surveyed individuals in 2016-2017 (33). Overweight and obesity were further associated with poorer glycemic control and more disturbed lipid profile, thereby increasing the cardiovascular risk (52, 53, 55). This cmorbid association highlights the need for targeted interventions focusing on weight control and metabolic risk reduction are essential to improve glycemic control and reduce the overall T2DM burden.

Hypertension and dyslipidemia were also associated with T2DM (30, 46, 47, 53). Furthermore, while T2DM prevalence increases dramatically in hypertensive patients, reaching 46%, this association is compounded by a poorer cardiac function (35, 63). The strong association between hypertension and T2DM, along with its impact on cardiac function, highlights the importance of systematic cardiovascular screening and early intervention.

Another comorbid association that is worth mentioning is depression (51). However, psychiatric comorbidity and psychological wellbeing constitute an unexplored area among Algerian patients with diabetes. This suggests an urgent need for further research and the incorporation of psychological assessments into diabetes care.

Addressing these comorbidities through multidisciplinary strategies will not only enhance patient outcomes but also reduce the overall healthcare burden of T2DM in Algeria.

### 4.5 Genetic and environmental factors

Data regarding genetic and environmental factors of diabetes in Algeria are very scarce, representing an extensive area for exploration via clinical, epidemiological, and environmental research. A study by Ouhaibi et al. explored the polymorphism of TCF7L2rs7903146 among a sample of 787 individuals and found that the T allele of the rs7903146 single nucleotide was associated with a 1.55-fold risk of T2DM. Additionally, the study identified a gene-diet interaction, where high dessert and milk intake further increased the risk of T2DM among T allele carriers (50). Another case-control study found up to 12.6-fold risk of diabetes associated with chlorinated persistent organic pollutants (59).

## 5 Diabetes management indicators in Algeria

### 5.1 Advances in diabetes care since 1962

In an interesting review published in a local medical journal ‘*Les Cahier du Praticien’*, Belhaj provided insights on the progression of diabetes management in Algeria, over the 60 years following the independence (64). The number of healthcare facilities has increased, reaching 15 university hospital centers and numerous public hospitals. This was associated with a substantial expansion of the private sector, where over 70% of patients with diabetes received treatment. Specialized private clinics have become crucial in diabetes management, suggesting potential for public-private collaboration. The country has also progressed in training diabetologists, although challenges like the ‘brain drain’ phenomenon persist. Efforts in continuing medical education have shown improvements in physicians’ knowledge and practices. Therapeutically, several improvements have been achieved along with advances in monitoring techniques like HbA1c measurement. However, the adoption of more recent therapies like insulin pumps and innovative medications has been slow, often hindered by high costs and lack of social security coverage.

### 5.2 Progression of prescribing practices

Very few publications explored the prescribing practices in T2DM in Algeria. This lack of data prevented from identifying accurate patterns and trends. The 2010 DiabCare series showed that approximately 45% of T2DM were on insulin, either alone (20%) or in association with an oral hypoglycemic drug (OHD, 26%). The most prescribed OHDs were biguanides (63.5%), sulphonylureas (SU) (44%), and meglitinides (6.7%). On the other hand, lifestyle and dietary measures were prescribed in 63% of the patients (46). The nationwide Barometer study (2013-2017) showed the predominance of OHD (85.4%), both in mono (44.3%) and bi-therapy (50.3%). The combination of metformin (Met) with insulin secretagogues (SU or glinides) was the most common (46.4%). Authors noted that no patient received dipeptidyl peptidase IV (DPP-4) inhibitors, glucagon-like peptide-1 receptor agonists (GLP-1 RA), or sodium/glucose cotransporter 2 (SGLT2) inhibitors (21). Another interesting insight was provided by baseline data from the DISCOVER study, an international prospective study launched in 2014, which involved 293 Algerian patients with diabetes recruited from 14 different sites nationally. The patterns of first- and second-line treatments were examined. In terms of first line therapy, the majority of patients (77.5%) received Met as a monotherapy, a small proportion (3.8%) received SU alone, while approximately 17% received a combination of Met with SU or another drug. In second line therapy, the most commonly prescribed treatment was Met+SU (35.8%), followed by Met + another medication (27.6%). Of note, 8.5% of patients were administered insulin, which could also be accompanied by oral therapy (65). In 2016, Ferdi et al. confirmed that the most common treatment prescribed for T2DM in Algeria was Met (65%) (54). It is worth noting that the prescribing practice is conditioned by the availability and reimbursement of the drugs.

### 5.3 Efficacy and safety data

Besides the paucity of studies, majority of real-world efficacy and safety data in Algeria concerned insulin. According to local practice, insulin is generally started in case of poor glycemic control or microvascular complications (66). Initiation of long-acting insulin among insulin-naïve patients on OHD failure improved glycemic control and reduced HbA1c levels, while mitigating hypoglycemia events (67). Similarly, the conversion from human to analogous insulin enabled better glycemic control and reduction of hypoglycemia events (68). In 2013, several sub-analyses studies of the A1chive project demonstrated the safety of insulin among Algerian T2DM patients and its effectiveness in improving glycemic control using various regimens (69–71). In 2015, an open-label multicenter randomized trial, Malek et al. compared the effectiveness of stepwise insulin intensification using basal-bolus insulin analogues (insulin detemir and aspart) versus biphasic insulin aspart 30 in insulin-naive T2DM patients in failure of OHDs. After 50 weeks of treatment, reductions in HbA1c levels were similar in the two regimens, as well as the incidence of hypoglycemia and other safety variables (72).

Furthermore, in Muslim countries like Algeria, the prescribing and monitoring of insulin pose further efficacy and safety concerns during Ramadan fasting. A multinational randomized trial compared the efficacy and safety of insulin degludec/insulin aspart (IDegAsp) with biphasic insulin aspart 30 (BIAsp 30) in patients with T2DM who fasted during Ramadan. Findings suggested that IDegAsp presents a lower risk of hypoglycemia for patients requiring insulin during Ramadan and beyond (73).

### 5.4 Levels of diabetes control

In 2004, severe outcomes were frequent among patients with uncontrolled diabetes, notably upper extremity infections, leading to death or amputations in many cases (44). Subsequent reports consistently showed inadequate glycemic control. In the DiabCare series, only 19% of the patients reached the target HbA1C (46). Data from the Barometer study (2013-2017) showed that as high as 64.6% of the patients had poor glycemic control indicated by HbA1c ≥7% and 20.1% had levels above 9% (21). More recent data, by Rahmoun et al. (74), showed an average blood glucose level of 206 mg/dL, with an average HbA1c of 8.2%, indicating suboptimal control (74). A similar trend was observed in female patients with diabetes, with Behar et al. (57) reporting an average HbA1c of 8.40% (57). Hacene et al. emphasized that non-adherence to insulin and poor self-monitoring of blood glucose (SMBG) practices were significant contributors to poor glycemic control (75).

### 5.5 Treatment compliance, self-management and patients’ education

Non-adherence rates of 31.3% and 36.5% were documented in studies conducted by (76) in 2019 and Hacene et al. in 2022, respectively (75, 76). Several factors were incriminated, such as lack of health insurance, poor self-monitoring, and disease duration of over six years. Consistently, two-third of the patients are reported to practice SMBG (46, 77). However, a national survey by the WHO (2016-2017) suggested lower adherence to regular SMBG, with only half of the patients having ever measured their blood glucose (78).

Lifestyle management and patient education are areas that need significant research attention. A study by Thanopoulou et al. (79) found that only a small fraction of Algerian T2DM patients meet nutritional or exercise recommendations (79). From another perspective, Belheddad and Azzoug (80) found that around half of T2DM patients received pre-Ramadan diabetes self-management education (80).

Finally, the use of traditional medicine is reported to be a common practice among T2DM patients in Algeria, requiring particular attention (81, 82), raising concerns of adverse herb-drug interactions (83).

## 6 Health and economic burdens of T2DM in Algeria

### 6.1 Health status at diagnosis as a reflection of diagnosis earliness

The health status of T2DM patients in Algeria was assessed in the large-scale screening campaign by Malek et al. (30). The prevalence of undiagnosed T2DM was 5.3%, associated with high frequencies of complications and comorbidities such as renal failure (15%), retinopathy (12.5%), and peripheral neuropathy (10%). Additionally, 11% of these individuals had abnormal EKG and comorbid dyslipidemia, hypertension, and obesity were found in more than 50% of these patients (30). Another interesting study showed that ketosis, diabetes foot, and cardiovascular events were the presenting signs for diabetes in 15.3%, 1.5% and 3.4% of the patients. Additionally, estimation of the cardiovascular risk showed that 36.7% had an intermediate and 37.7% had high-to-very high risk (84).

### 6.2 Hypoglycemia burden

The incidence of hypoglycemia events is high among Algerian T2DM patients. In 2019, Sellam et al. showed that non-severe nocturnal hypoglycemic events (NSNHEs), non-severe hypoglycemic events (NSHEs), and severe hypoglycemic events (SHEs) occurred at the respective rates of 9.8, 30.5, and 0.3 events per patient-year (85). More recently, Mimoune et al. (62) reported hypoglycemia events among 29.5% of the patients, with the main risk factors being insulin regimens combining basal insulin and rapid-acting insulin, along with absence of self-monitoring (77). Both severe and non-severe hypoglycemia events were demonstrated by the DAWN2 study to impact patients’ wellbeing and overall quality of life. Notably, participants who had experienced SHE reported more discrimination feelings because of their condition with diabetes; however, they acknowledged receiving higher levels of support for managing their condition (86).

### 6.3 Other diabetes complications

Regarding the other complications of T2DM, the available data is highly heterogeneous, probably due to the varying stage at which patients were assessed, along with the variability in the diagnostic criteria. The reported frequency of diabetic retinopathy varies from 12.5% to 68% (30, 46, 58, 87, 88), while that of neuropathy varies from 2.8% to 46.2% (30, 46, 58, 61, 62, 87, 89), while diabetic nephropathy was reported in up to 7.5% of the patients (58, 87). In evaluating the cardiovascular risk, Boukheloua et al. identified diabetes as an independent factor for severe coronary lesion with an OR of 1.92 (90).

Mimoune et al. (62) evaluated peripheral arterial disease in 47 T2DM patients in Constantine. They observed a high prevalence of peripheral artery disease (80.85%) with various presentations, such as obliterating arteriopathy of the lower limbs (57.45%), media calcinosis (42.55%), and acute arterial occlusion (21.28%). The study also reported a neuropathy prevalence of 46.81%, and all the patients (100%) presented varying stages of diabetic foot (62). Notwithstanding the methodological limitations of the previous study, such complications are likely to be more prevalent in patients with inadequate glycemic control. In a series by Ayad et al. (87), which involved 310 patients (172 T2DM and 138 T1DM) with poor glycemic control, the frequency of coronary artery disease and retinopathy was reported equally in 20% of the patients, besides a high frequency of peripheral neuropathy (42.5%) (87). Another interesting data is reported by Belhadj et al., which included 1,974 T2DM patients who were inadequately controlled on OHDs. Authors reported as high as 59.7% frequency of macrovascular complications, of which 6.8% were coronary artery disease, noting that 31% of the patients had at least one comorbidity (67).

Published data consistently point towards a high prevalence of peripheral neuropathy and neuropathic pain among Algerian patients with diabetes. Malek et al. (30) reported a striking 75% prevalence of neuropathy (30), which is consistent with data by Aouiche et al. (89) reporting a prevalence of 68% (89). However, Maamar et al. (91), reported a lower prevalence of peripheral neuropathy of 46.2% of the population with diabetes (91). This complication is associated with a high risk of neuropathic pain, as demonstrated by Ayad et al. (87), who highlighted painful neuropathy in 46% of the patients (87). This is corroborated by Nibouche-Hattab’s data showing a painful neuropathy among 45% of newly diagnosed T2DM patients (84).

Regarding diabetic foot, Benotmane et al. (44) found a 26.4% rate of microvascular complications, often a precursor to diabetic foot (44). Conversely, Zaoui S et al. (24) analyzed a larger demographic in Tlemcen, reporting an 8.2% occurrence of foot lesions and 0.7% of amputations (24). A higher prevalence (22.15%) was observed by Bounihi et al. in Algiers, albeit in a controlled sample of 130 individuals (58). In contrast, Ayad et al. (87), recorded the lowest prevalence of 1.5% among a larger sample in Oran, focusing on poorly controlled patients with diabetes (87).

### 6.4 Mortality data

According to the Global Burden of Disease data, T2DM was associated with 12.73 per 100,000 deaths in Algeria, in 2019. Additionally, between 1990 and 2019, T2DM rank passed from 17^th^ to the 9^th^ rank of mortality causes in the Algerian population (92). No further T2DM associated mortality data are found in the Algerian literature.

### 6.5 Economic burden

Accurate estimates of T2DM economic costs in Algeria are yet to be determined. One study has estimated the national economic burden of T2DM at 45 Bn DZD (60 060 DZD per patient), 58% of which was spent on complications’ management while the medication costs accounted for 34% (25). Another study by Sellam et al. estimated the economic cost of insulin-related hypoglycemia in Algerian adults with T1DM and T2DM at DZD 42.9 billion (USD 334 million), with T2DM accounting for 74%. Of the total, direct costs were estimated to be DZD 8.73 million (USD 68.1 million), while indirect costs related to productivity loss were DZD 34.2 billion (USD 265.6 million). This translated to a total 4,769,874 patient-productivity days lost (85). No further data was found regarding the economic burden of T2DM in Algeria.

## 7 Discussion

### 7.1 Lessons learned from epidemiological data

We observed a great heterogeneity in the methodologies employed in epidemiological studies, which introduces variability in reported prevalence rates. However, figures are obviously on the rise, as demonstrated by the WHO StepWise surveys showing an increase from 8.9% in 2003 to 14.4% in 2016-2017 (27, 33), a growth of almost 75%. Compared to the other MENA countries, Algeria was ranked 14^th^ in terms of T2DM prevalence, in 2014 (93). In the African continent, data from 1980-2014 show a substantial increase in the age-standardized prevalence of adult diabetes and BMI in all countries, including Algeria. By focusing on 2014, Algeria’s figures were the highest compared to the other African countries (94). On the other hand, the prevalence trends observed in our data compare relatively well with those available in the Global Burden of Disease database (GBD) (92). We note, however, lower figures (roughly 7% versus 15%) in the GBD estimates for the period 2015-2020, compared with our data, respectively. This low rate is inconsistent with the findings from the second StepWise survey, which found a prevalence of 14.40% (33), indicating that our estimates are more accurate.

It is worth mentioning that Algeria, among other emerging countries, is undergoing demographic and lifestyle changes that potentially escalate the risk of T2DM. Predictive models forecast a substantial rise in T2DM prevalence in the coming decades in Algeria and other MENA countries. The number of people with diabetes is estimated to rise by 143% in Africa and 96% in the MENA region by 2045, representing the highest trends globally (95). From a public health perspective, this increase underscores the need for Algeria and its MENA counterparts to prioritize diabetes prevention and management strategies. This predicts an imminent strain on healthcare infrastructure and resources, requiring proactive policy measures and investments in health systems to cope with the anticipated demand. Furthermore, these findings underscore the importance of regional collaboration to address the growing burden of the disease.

Nevertheless, we note an important discrepancy in the estimates, especially across the different population-based studies. This is more notable considering the very low prevalence rates (3.9% among adults) reported by the 2013 MOH-UNICEF- UNFPA study (31), which are inconsistent with the trending rates in the same period.

### 7.2 Profiling the Algerian T2DM patient

The present systematic analysis revealed several socioeconomic determinants and risk factors associated with T2DM in Algeria. Diabetes onset typically presents in an overweight, quadragenarian female or male, likely having poor dietary and lifestyle habits and accumulating other metabolic and cardiovascular risk factors. Low economic class and lower education, besides lack of health insurance registration, further contribute to increased T2DM risk among disadvantaged groups, exacerbating health inequities. Comorbidities such as depression and genetic and environmental factors are poorly explored and appear to play a role in the disease. Considering these factors are vital in elaborating targeted interventions to effectively combat T2DM and its associated burdens among the Algerian population. Furthermore, due to the scarcity of data and limited research locally, there is a pressing need for further investigation to comprehensively understand the risk factors of T2DM in Algeria.

### 7.3 Opportunities to improve T2DM management

The present project aims to propel the implementation of a national plan for the primary prevention of diabetes. Thanks to governments’ efforts, including continuing medical education and screening programs, the number of undiagnosed patients has significantly decreased over the past decades. Nevertheless, there remains a critical need to promote secondary and tertiary prevention, as well as management and screening of comorbidities associated with diabetes (30, 84, 96–98). This is evidenced by the significant health and economic burdens associated with the complications of diabetes (24, 25, 30, 46, 58, 61, 62, 87, 89–91). That being said, an effective approach involves a multi-faceted strategy that encompasses enhancing care delivery, patient education, and public awareness, as well as improving access to new multipotent drugs.

Prevention strategies should be strengthened through systematic screening programs in primary care centers, pharmacies, and workplaces, targeting high-risk populations. Secondary and tertiary prevention should also be considered. Standardized national guidelines for early detection and management of complications such as neuropathy, nephropathy, and cardiovascular disease are essential to reduce long-term morbidity. Additionally, integrating multidisciplinary care teams, including endocrinologists, cardiologists, nephrologists, ophthalmologists, and nutritionists, can enhance comprehensive diabetes management and improve patient outcomes.

Patient education should be a priority action through structured diabetes education programs that can be delivered via community-based and or digital platforms, focusing on lifestyle modification, medication adherence, and self-monitoring. Culturally tailored educational materials in Arabic and Berber languages, incorporating local dietary habits, can enhance patient engagement and compliance. Additionally, expanding peer support networks and involving community health workers in self-care education can further empower patients in managing their condition effectively.

Furthermore, our review of prescribing practices revealed underutilization of some hypoglycemic agents, particularly those in the SGLT2 inhibitor and GLP-1 receptor agonist classes (21, 54, 65). The application of international recommendations advocating prescribing practices tailored to the patient’s profile is essential. However, to sustain this measure, it is crucial to ensure broader coverage of modern antidiabetic medications with proven cardiovascular and renal benefits. It is also relevant to consider health insurance policy reform, focusing on the role of private insurance in bridging the gap in the universal health coverage system and improving access to treatment (19, 21, 99).

Another measure to improve the management of T2DM is to encourage the homogenization and accreditation of continuing education of healthcare professionals, notably to improve practices and foster multidisciplinary care (100). The involvement and training of community healthcare workers in diabetes management is increasingly recommended to enhance case detection and glycemic control and reduce cardiovascular morbidity, especially in underserved areas (101).

These measures necessitate a data-driven and research-based strategy, coupled with a patient-centered approach to optimize their impact on patients’ physical and overall wellbeing (102–104). Such comprehensive strategy should yield evidence-based recommendations specifically tailored to the Algerian context for optimal results.

### 7.4 Addressing knowledge gap to further improve T2DM management

A major limitation in T2DM research in Algeria is the high heterogeneity in methodologies used and the evident absence of incidence data, which is critical for predicting future disease burden, optimizing resource allocation, and designing targeted interventions for high-risk populations. Moreover, the lack of systematic data on the interplay of these determinants with genetic and environmental determinants hinders the development of tailored prevention strategies. Without such data, it is difficult to monitor the impact of preventive measures and address health inequalities and other social determinants of diabetes. Addressing these gaps requires comprehensive, population-based studies that explore the multifactorial nature of T2DM in Algeria.

Furthermore, mortality data specific to T2DM in Algeria are largely absent, with available estimates relying on the Global Burden of Disease database (92). Similarly, the economic burden of T2DM is inadequately quantified, likely leading to an underestimation of its healthcare and societal costs. Understanding these aspects through well-designed epidemiological and health economics studies is crucial for improving T2DM care and policy formulation.

The clinical data of T2DM management in Algeria also has significant limitations. There is a lack of real-world evidence on the efficacy and safety of antidiabetic treatments, especially OHDs and newer antidiabetic agents, limiting the ability to optimize treatment strategies based on local patient populations. Additionally, data on treatment outcomes, including glycemic control, adherence, and self-management, are scarce, making it difficult to assess the effectiveness of current management approaches. Similarly, data on adherence and efficacy of lifestyle interventions have not been sufficiently studied in Algeria, with an absence of interventional and long-term cohort studies evaluating their impact. Finally, the available data on T2DM complications are heterogeneous, likely due to variability in diagnostic criteria and differences in disease stage at the time of assessment.

Addressing these knowledge gaps requires comprehensive epidemiological and clinical research initiatives. National registries, longitudinal cohort studies, and real-world treatment outcome assessments are essential to generating reliable data for guiding public health policies and improving patient care. Strengthening research infrastructure and fostering collaboration between healthcare institutions, academic centers, and policymakers will be key to advancing T2DM management in Algeria.

## 8 Conclusion

This comprehensive review on T2DM care in Algeria reveals a multifaceted challenge. Despite the increasing burden of T2DM, the country’s healthcare approach remains suboptimal. The escalating prevalence of diabetes and obesity in Algeria underscores an urgent need for action. Immediate attention is required, as current data and alarming future predictions emphasize the necessity of effective preventive strategies. Primary prevention programs are imperative to counteract this growing pandemic, necessitating strong political commitment to promote significant lifestyle modifications, with a particular focus on nutrition, physical activity, and widespread awareness.

To optimize diabetes management in Algeria, a comprehensive transformation of healthcare organization is imperative. This entails enhancing prescription practices through continuous education for healthcare professionals. Digitizing medical records is crucial for improved patient management, while optimizing resource utilization requires defining clear priorities and objectives. Additionally, providing personalized and rational access to new therapies, despite their high costs, is essential. Supporting these initiatives necessitates diversifying healthcare financing sources, potentially integrating private insurance, mutual funds, and workers’ social funds. This approach ensures equitable access to healthcare and treatments, fostering a more effective and sustainable diabetes care system.

## References

[1] Khan MAB Hashim MJ King JK Govender RD Mustafa H Al Kaabi J. Epidemiology of type 2 diabetes – global burden of disease and forecasted trends. J. Epidemiol. Glob Health. (2019) 10:107. doi: 10.2991/jegh.k.191028.001 PMC7310804 32175717

[2] Sun H Saeedi P Karuranga S Pinkepank M Ogurtsova K Duncan BB. IDF Diabetes Atlas: Global, regional and country-level diabetes prevalence estimates for 2021 and projections for 2045. Diabetes Res. Clin. Pract. (2022) 183:109119. doi: 10.1016/j.diabres.2021.109119 34879977 PMC11057359

[3] Safiri S Karamzad N Kaufman JS Bell AW Nejadghaderi SA Sullman MJM. Prevalence, deaths and disability-adjusted-life-years (DALYs) due to type 2 diabetes and its attributable risk factors in 204 countries and territories 1990-2019: results from the global burden of disease study 2019. Front. Endocrinol. (Lausanne). (2022) 13:838027. doi: 10.3389/fendo.2022.838027 35282442 PMC8915203

[4] Chaturvedi N. The burden of diabetes and its complications: Trends and implications for intervention. Diabetes Res. Clin. Pract. (2007) 76:S3–S12. doi: 10.1016/j.diabres.2007.01.019 17343954

[5] Yahagi K Kolodgie FD Lutter C Mori H Romero ME Finn AV. Pathology of human coronary and carotid artery atherosclerosis and vascular calcification in diabetes mellitus. Arterioscler. Thromb. Vasc. Biol. (2017) 37:191–204. doi: 10.1161/ATVBAHA.116.306256 27908890 PMC5269516

[6] Wang M-C Li C-I Liu C-S Lin C-H Yang S-Y Li T-C. Effect of blood lipid variability on mortality in patients with type 2 diabetes: a large single-center cohort study. Cardiovasc. Diabetol. (2021) 20:228. doi: 10.1186/s12933-021-01421-4 34823536 PMC8620132

[7] DeFronzo RA. From the triumvirate to the ominous octet: A new paradigm for the treatment of type 2 diabetes mellitus. Diabetes. (2009) 58:773–95. doi: 10.2337/db09-9028 PMC2661582 19336687

[8] D’Alessio D. The role of dysregulated glucagon secretion in type 2 diabetes. Diabetes Obes. Metab. (2011) 13:126–32. doi: 10.1111/j.1463-1326.2011.01449.x 21824266

[9] Deacon CF Ahrén B. Physiology of incretins in health and disease. Rev. Diabetic Stud. (2011) 8:293–306. doi: 10.1900/RDS.2011.8.293 22262068 PMC3280665

[10] Galicia-Garcia U Benito-Vicente A Jebari S Larrea-Sebal A Siddiqi H Uribe KB. Pathophysiology of type 2 diabetes mellitus. Int. J. Mol. Sci. (2020) 21:6275. doi: 10.3390/ijms21176275 32872570 PMC7503727

[11] Sears B Perry M. The role of fatty acids in insulin resistance. Lipids Health Dis. (2015) 14:121. doi: 10.1186/s12944-015-0123-1 26415887 PMC4587882

[12] Lee S-H Park S-Y Choi CS. Insulin resistance: from mechanisms to therapeutic strategies. Diabetes Metab. J. (2022) 46:15–37. doi: 10.4093/dmj.2021.0280 34965646 PMC8831809

[13] Shanik MH Xu Y Skrha J Dankner R Zick Y Roth J. Insulin resistance and hyperinsulinemia: is hyperinsulinemia the cart or the horse? Diabetes Care. (2008) 31 Suppl 2:S262–8. doi: 10.2337/dc08-s264 18227495

[14] Samuel VT Shulman GI. The pathogenesis of insulin resistance: integrating signaling pathways and substrate flux. J. Clin. Invest. (2016) 126:12–22. doi: 10.1172/JCI77812 26727229 PMC4701542

[15] Shpakov AO Derkach KV Berstein LM. Brain signaling systems in the Type 2 diabetes and metabolic syndrome: promising target to treat and prevent these diseases. Future Sci. OA. (2015) 1:FSO25. doi: 10.4155/fso.15.23 28031898 PMC5137856

[16] Nespoux J Vallon V. Renal effects of SGLT2 inhibitors: an update. Curr. Opin. Nephrol. Hypertens. (2020) 29:190–8. doi: 10.1097/MNH.0000000000000584 PMC7224333 31815757

[17] Kanie T Mizuno A Takaoka Y Suzuki T Yoneoka D Nishikawa Y. Dipeptidyl peptidase-4 inhibitors, glucagon-like peptide 1 receptor agonists and sodium-glucose co-transporter-2 inhibitors for people with cardiovascular disease: a network meta-analysis. Cochrane Database Syst. Rev. (2021) 10:CD013650. doi: 10.1002/14651858.CD013650.pub2 34693515 PMC8812344

[18] Ni X Zhang L Feng X Tang L. New hypoglycemic drugs: combination drugs and targets discovery. Front. Pharmacol. (2022) 13:877797. doi: 10.3389/fphar.2022.877797 35865956 PMC9295075

[19] ElSayed NA Aleppo G Aroda VR Bannuru RR Brown FM Bruemmer D. Obesity and weight management for the prevention and treatment of type 2 diabetes: standards of care in diabetes-2023. Diabetes Care. (2023) 46:S128–39. doi: 10.2337/dc23-S008 36507637 PMC9810466

[20] Chibane A Attif ML Makhlouf L Lanasri N Biad A. P110 Prévalence du diabète de type 2 dans une banlieue d’Alger. Diabetes Metab. (2008) 34:H74. doi: 10.1016/S1262-3636(08)73022-2

[21] Belhadj M Arbouche Z Brouri M Malek R Semrouni M Zekri S. BAROMÈTRE Algérie: enquête nationale sur la prise en charge des personnes diabétiques. Méd Des. Maladies Métaboliques. (2019) 13:188–94. doi: 10.1016/S1957-2557(19)30055-0

[22] Carretero-Anibarro E Hamud-Uedha M. Prevalence of undiagnosed type 2 diabetes mellitus in the sahrawi population of the sahrawi refugee camps of Tindouf, Algeria. Med. Clin. (Barc). (2020) 155:461–2. doi: 10.1016/j.medcli.2019.05.035 31427152

[23] Moussouni A Sidi-yakhlef A Hamdaoui H Aouar A Belkhatir D. Prevalence and risk factors of prehypertension and hypertension in Algeria. BMC Public Health. (2022) 22:1571. doi: 10.1186/s12889-022-13942-y 35982441 PMC9386961

[24] Zaoui S Biémont C Meguenni K. Epidemiology of diabetes in urban and rural regions of Tlemcen (Western Algeria). Sante. (2007) 17:15–21. 17897897

[25] Oulad abdallah R Boukana R Loumi W Nebchi E Mimene A. Type 2 diabetes mellitus in Algeria – a cost of illness study. Value Health. (2018) 21:S126. doi: 10.1016/j.jval.2018.09.754

[26] Malek R. [amp]]Eacute;pidémiologie du diabète en Algérie: revue des données, analyse et perspectives. Méd Des. Maladies Métaboliques. (2011) 5:29–33. doi: 10.1016/S1957-2557(11)70069-4

[27] World Health Organization (WHO) Direction de la Prévention - Ministry of Health. (2004). Mesure des facteurs de risque des maladies non transmissibles dans deux zones pilotes (Approche StepWise).

[28] Institut National de Santé Publique (INSP). Transition épidémiologique et système de santé: Projet TAHINA. Algiers. (2007). doi: 10.13140/RG.2.1.1668.4967

[29] Ministry of Health Office National des Statistiques. Multiple Indicator Cluster Survey (MICS 3) (2008). Available online at: https://mics-surveys-prod.s3.amazonaws.com/MICS3/MiddleEastandNorthAfrica/Algeria/2006/Final/Algeria2006MICS_French.pdf (Accessed September 10, 2024).

[30] Malek R NeChadi A Rezig M-F Abdelaziz S Mallem N Bouferroum A. Dépistage de masse du diabète de type 2 en Algérie: quels enseignements? Méd Des. Maladies Métaboliques. (2013) 7:557–62. doi: 10.1016/S1957-2557(13)70714-4

[31] Ministry of Health UNICEF UNFPA. Multiple Indicator Cluster Survey (MICS 4) 2012-2013 (2015). Available online at: https://www.unicef.org/Algeria/sites/unicef.org.Algeria/files/2018-04/RapportMICS4%282012-2013%29.pdf (Accessed September 10, 2024).

[32] Handlos LN Witte DR Almdal TP Nielsen LB Badawi SE Sheikh ARA. Risk scores for diabetes and impaired glycaemia in the Middle East and North Africa. Diabetes Med. (2013) 30:443–51. doi: 10.1111/dme.12118 23331167

[33] World Health Organization. Enquête nationale sur la mesure du poids des facteurs de risque des Maladies Non Transmissibles selon l’approche STEPwise de l’OMS Principaux résultats (2017). Available online at: https://www.afro.who.int/sites/default/files/2018-12/fasciculeresultatssteps14novembre2018_1.pdf (Accessed September 10, 2024).

[34] Houti L. Epidémiologie du diabète de type 2 chez l’adulte âgé de 30 à 64 ans dans la wilaya d’Oran: Approche méthodologique. Diebetes Int. (2001) 11:4–8.

[35] Malek R Belateche F Laouamri S Hamdi-Cherif M Touabti A Bendib W. Prevalence of type 2 diabetes mellitus and glucose intolerance in the Setif area (Algeria). Diabetes Metab. (2001) 27:164–71. 11353884

[36] Kemali Z Hanaizi H Kara B Kanoun N Kemali N Ferrah T. Le diabète sucré et ses facteurs de risque dans une population adulte. Rev. Algérienne Santé Militaire. (1995) 24:7–14.

[37] Bezzaoucha A. Le diabète sucré connu à Alger: fréquence et conséquences. Diabetes Metab. (1992) 18:229–35. 1397478

[38] Belhadj M Ousidhoum M Midoune N Cherrak A Aribi S Bachaoui M. The prevalence of Type 2 diabetes mellitus in Touaregs of South Algeria. Diabetes Metab. (2003) 29:298–302.

[39] Yahia-Berrouiguet A Benyoucef M Meguenni K Brouri M. Enquête sur la prévalence des facteurs de risque de maladies cardiovasculaires à Tlemcen (Algérie). Méd Des. Maladies Métaboliques. (2009) 3:313–9. doi: 10.1016/S1957-2557(09)74761-3

[40] Chami M-A Zemmour L Midoun N Belhadj M. Diabète sucré du sujet âgé: la première enquête algérienne. Méd Des. Maladies Métaboliques. (2015) 9:210–5. doi: 10.1016/S1957-2557(15)30046-8

[41] Dalichaouche-Benchaoui S Abadi N. Association between obesity and metabolic anomalies in a population in eastern Algeria. Jordan Med J. (2022) 56. doi: 10.35516/jmj.v56i3.361

[42] Azzouz M Boudiba A Guerchani M-K Lyes Y Hannachi R Baghous H. Apport du score de risque Finlandais FINDRISC dans l’identification de la dysglycémie dans une population algéroise, Algérie. Méd Des. Maladies Métaboliques. (2014) 8:532–8. doi: 10.1016/S1957-2557(14)70877-6

[43] Nebab A Arbouche Z Ayoub S Nadir D Tebaibia A. [amp]]Eacute;tude observationnelle sur la fréquence du surpoids et de l’obésité chez les patients âgés de 18 ans et plus, vus en consultation de médecine générale, diabétologie, médecine interne et endocrinologie des secteurs public et privé en Algérie. Rev. Algérienne Méd Interne. (2022) 12:33–7. https://ramidz.com/RAMIDZ/index.php/RAMI/article/view/40 (Accessed September 10, 2024).

[44] Benotmane A Faraoun K Mohammedi F Benkhelifa T Amani ME. Infections of the upper extremity in hospitalized diabetic patients: A prospective study. Diabetes Metab. (2004) 30:91–7. doi: 10.1016/S1262-3636(07)70094-0 15029103

[45] Houti L Ouhaibi-Djellouli H Hamani-Medjaoui I Lardjam-Hetraf S Goumidi L Mediene-Benchekor S. CA-082: Épidémiologie du diabète de type 2 et ses facteurs de risque dans la population adulte oranaise (Étude ISOR). Diabetes Metab. (2016) 42:A57. doi: 10.1016/S1262-3636(16)30214-2

[46] Belhadj M Malek R Boudiba A Lezzar E Roula D Sekkal F. DiabCare algérie: diabCare Algeria. Méd Des. Maladies Métaboliques. (2010) 4:88–92. doi: 10.1016/S1957-2557(10)70020-1

[47] Belmokhtar F Belmokhtar R Charef M. Risk factors associated with type 2 diabetes mellitus in west region of Algeria, Maghnia. J. Diabetes Metab. (2011) 02:2. doi: 10.4172/2155-6156.1000148

[48] Sahi MD Metri AA Belmokhtar F Belmokhtar R Boazza F. The relationship between ABO/rhesus blood groups and type 2 diabetes mellitus in maghnia, western algeria. . South Afr Family Pract. (2011) 53:6:568–72. doi: 10.1080/20786204.2011.10874154

[49] Mohammed Nadjib R Amine G Amine HM. Glycated hemoglobin assay in a Tlemcen population: Retrospective study. Diabetes Metab. Syndrome: Clin. Res. Rev. (2018) 12:911–6. doi: 10.1016/j.dsx.2018.05.014 29802072

[50] Ouhaibi-Djellouli H Mediene-Benchekor S Lardjam-Hetraf SA Hamani-Medjaoui I Meroufel DN Boulenouar H. The TCF7L2rs7903146 polymorphism, dietary intakes and type 2 diabetes risk in an Algerian population. BMC Genet. (2014) 15:134. doi: 10.1186/s12863-014-0134-3 25491720 PMC4266211

[51] Sebbani M Dali Sahi M Amine M Aouar A. Prévalence du diabète de type 2 et facteurs associés au cours de la dépression. Encephale. (2014) 40:9–14. doi: 10.1016/j.encep.2013.03.006 23816058

[52] Diaf M Khaled BM Habib HH Belbraoued S. Effect of gender and body weight on postprandial glucose and lipid metabolism in adults with type 2 diabetes. JNMA J. Nepal Med. Assoc. (2014) 52:866–77. 26982659

[53] Diaf M Khaled MB Sellam F. Correlation between dietary fat intake and atherogenic indices in normal, overweight and obese adults with or without type 2 diabetes. Rom J. Diabetes Nutr. Metab. Dis. (2015) 22:347–60. doi: 10.1515/rjdnmd-2015-0041

[54] Ferdi NEH Abla K Chenchouni H. Effect of socioeconomic factors and family history on the incidence of diabetes in an adult diabetic population from Algeria. Iran J. Public Health. (2016) 45:1636–44. PMC5207105 28053930

[55] Ferdi NEH Abla K Chenchouni H. Biochemical profile of an adult diabetic population from Algeria in relation with anthropometric parameters, age and gender. Iran J. Public Health. (2018) 47:1119–27. PMC6123603 30186783

[56] Kachekouche Y Dali-Sahi M Benmansour D Dennouni-Medjati N. Hematological profile associated with type 2 diabetes mellitus. Diabetes Metab Syndrome: Clin Res Rev. (2018) 12(3):309–12. doi: 10.1016/j.dsx.2017.12.015 29287841

[57] Behar A Dennouni-Medjati N Dali-Sahi M Harek Y Belhadj M Benslama Y. Dietary selenium intake and risk of type 2 diabetes in a female population of western Algeria. Nutr. Clinique Métabol. (2020) 34:254–8. doi: 10.1016/j.nupar.2020.04.005

[58] Bounihi A Saidi H Bouazza A Benbaibeche H Azzouz M Koceir EA. Dietary diversity as a risk factor for obesity in Algerian patients with type 2 diabetes mellitus. Healthcare (Basel). (2021) 9:1229. doi: 10.3390/healthcare9091229 34575003 PMC8468535

[59] Mansouri EH Reggabi M. Association between type 2 diabetes and exposure to chlorinated persistent organic pollutants in Algeria: A case-control study. Chemosphere. (2021) 264:128596. doi: 10.1016/j.chemosphere.2020.128596 33059283

[60] Khaldi Z Nayme K Bourjilat F Bensaci A Timinouni M El-Hadj-Khelil AO. Detection of ESBLs and carbapenemases among enterobacteriaceae isolated from diabetic foot infections in ouargla, algeria. J Infection Developing Countries. (2022) 16(11):1732–8. doi: 10.3855/jidc.16660 36449645

[61] Hafidh K Malek R Al-Rubeaan K Kok A Bayram F Echtay A. Prevalence and risk factors of vascular complications in type 2 diabetes mellitus: Results from discover Middle East and Africa cohort. Front. Endocrinol. (Lausanne). (2022) 13:940309. doi: 10.3389/fendo.2022.940309 36017310 PMC9396276

[62] Mimoune H Debbache HSLN Derris A Roula D Kitouni Y. Study of peripheral arterial disease among type 2 diabetes mellitus patients. Diabetes Res. Clin. Pract. (2022) 186:109424. doi: 10.1016/j.diabres.2022.109424

[63] Soufi Taleb Bendiab N Ouabdesselam S Henaoui L Lopez-Sublet M Monsuez J-J Benkhedda S. Impact of diabetes on cardiac function in patients with high blood pressure. Int. J. Environ. Res. Public Health. (2021) 18:6553. doi: 10.3390/ijerph18126553 34207036 PMC8296398

[64] Belhadj M. 60 ans de Diabétologie Adulte en Algérie, (1962-2022). Les Cahiers du Praticien. (2022) 6:47–54. www.lcpramp.com (Accessed September 10, 2024).

[65] Malek R Brouri M Ammi M. Type 2 Diabetes and comorbidities DISCOVERing in Real World:(From The DISCOVER Program*): Algeria Baseline Data. Revue Algérienne de Médecine Interne. (2019) 9:21–8. Available at: https://ramidz.com/RAMIDZ/index.php/RAMI/article/download/55/53 (Accessed March 13, 2025).

[66] Malek R Arbouche Z Bachaoui M Zinai S Dahaoui A Senoussaoui S. Criteria influencing the choice of starting insulin regimen in patients with type 2 diabetes in routine clinical practice: baseline data from the Algerian cohort of the A1chieve study. Diabetes Res. Clin. Pract. (2013) 101:S45–9. doi: 10.1016/S0168-8227(13)70018-4 23958572

[67] Belhadj M Roula D Malek R Lezzar A Mimouni S Zinai S. Initiation de l’insuline détémir chez des patients diabétiques de type 2 insulino-naïfs en échec aux antidiabétiques oraux: étude de tolérance et d’efficacité en pratique courante en Algérie (Étude IDEALS). Méd Des. Maladies Métaboliques. (2012) 6:332–7. doi: 10.1016/S1957-2557(12)70424-8

[68] Arbouche Z Lezzar A Salah-Mansour A Zinai S. Le transfert des insulines humaines vers les analogues de l’insuline entraîne une amélioration de l’HbA1c et une réduction des hypoglycémies chez les patients diabétiques de type 2: données de la cohorte algérienne de l’étude A1chieve. Méd Des. Maladies Métaboliques. (2012) 6:511–8. doi: 10.1016/S1957-2557(12)70472-8

[69] Lezzar A Ayad F Dahaoui A Salah-Mansour A Berrouiguet AY. Initiating or switching to biphasic insulin aspart 30 in type 2 diabetes patients from Algeria: a sub-analysis of the A1chieve study. Diabetes Res. Clin. Pract. (2013) 101:S37–44. doi: 10.1016/S0168-8227(13)70017-2 23958570

[70] Malek R Arbouche Z Dahaoui A Bachaoui M. Safety and effectiveness of insulin analogues in type 2 diabetic patients from Algeria: a sub-analysis of the A1chieve study. Diabetes Res. Clin. Pract. (2013) 101:S15–26. doi: 10.1016/S0168-8227(13)70015-9 23958568

[71] Hajjaji I Shah S Li Y Prusty V Benabbas Y Home PD. Safety, tolerability, and efficacy of insulin aspart in people with type 2 diabetes, as biphasic insulin aspart or with basal insulin: findings from the multinational, non-interventional A1chieve study. Diabetes Ther. (2014) 5:113–26. doi: 10.1007/s13300-014-0052-4 PMC4065304 24477669

[72] Malek R Ajili F Assaad-Khalil SH Shinde A Chen JW Van den Berg E. Similar glucose control with basal–bolus regimen of insulin detemir plus insulin aspart and thrice-daily biphasic insulin aspart 30 in insulin-naive patients with type 2 diabetes: Results of a 50-week randomized clinical trial of stepwise insulin intensif. Diabetes Metab. (2015) 41:223–30. doi: 10.1016/j.diabet.2014.11.002 25483023

[73] Hassanein M Echtay AS Malek R Omar M Shaikh SS Ekelund M. Efficacy and safety analysis of insulin degludec/insulin aspart compared with biphasic insulin aspart 30: A phase 3, multicentre, international, open-label, randomised, treat-to-target trial in patients with type 2 diabetes fasting during. Diabetes Res. Clin. Pract. (2018) 135:218–26. doi: 10.1016/j.diabres.2017.11.027 29183844

[74] Rahmoun MN Ghembaza CE El-Amine-Ghembaza M. Lipid profile in type 2 patients with diabetes from Tlemcen: A Western Algerian population. Diabetes Metab. Syndrome: Clin. Res. Rev. (2019) 13:1347–51. doi: 10.1016/j.dsx.2019.02.008 31336490

[75] Hacene MNB Zatla YT Saker M Abbou Z Youcef A Boulenouar H. Factors associated with non-adherence to insulin in Type 1 and Type 2 diabetes mellitus patients in Western region of Algeria, Tlemcen: a cross-sectional study. Pan Afr. Med. J. (2022) 41:172. doi: 10.11604/pamj.2022.41.172.32972 35573427 PMC9074048

[76] Achouri MY Mammeri M Sehanine Y Selka MA Ghomari WI Lahmer A. Facteurs associés à la non-observance thérapeutique chez les diabétiques de type 2: première enquête algérienne. Ann. Pharm. Fr. (2019) 77:506–15. doi: 10.1016/j.pharma.2019.08.003 31564421

[77] Mimouni S Faraoun K Nouri N Serouti A. Fréquence de l’hypoglycémie chez les patients diabétiques de type 2 traités par insuline basale en Algérie (Hypo Study). Méd Des. Maladies Métaboliques. (2022) 16:351–8. doi: 10.1016/j.mmm.2022.04.005

[78] Ministry of Health. Algeria - STEPS 2016 . Available online at: https://extranet.who.int/ncdsmicrodata/index.php/catalog/91/data-dictionary/F10?file_name=dza2016 (Accessed June 23, 2023).

[79] Thanopoulou A Karamanos B Angelico F Assaad-Khalil S Barbato A Del Ben M. Nutritional habits of subjects with Type 2 diabetes mellitus in the Mediterranean Basin: comparison with the non-diabetic population and the dietary recommendations. Multi-Centre Study of the Mediterranean Group for the Study of Diabetes (MGSD). Diabetologia. (2004) 47:367–76. doi: 10.1007/s00125-003-1316-0 14730377

[80] Belheddad AZ Azzoug S. Ramadan fasting in a sample Algerian population with diabetes. Diabetes Res. Clin. Pract. (2022) 188:109901. doi: 10.1016/j.diabres.2022.109901 35513160

[81] Telli A Esnault M-A Ould El Hadj Khelil A. An ethnopharmacological survey of plants used in traditional diabetes treatment in south-eastern Algeria (Ouargla province). J. Arid Environ. (2016) 127:82–92. doi: 10.1016/j.jaridenv.2015.11.005

[82] Aissa L Zohra M El Houda HN Zohra DF. Ethnobotanical study of medicinal plants used for the treatment of Diabetes mellitus in Sidi Bel Abbes region (North-west Algeria). Bol Latinoam Caribe Plantas Med. Aromat. (2019) 18. doi: 10.35588/blacpma.19.18.4.25

[83] Chelghoum M Khitri W Bouzid S Lakermi A. New trends in the use of medicinal plants by Algerian diabetic patients, considerations of herb-drug interactions. J. Ethnopharmacol. (2021) 274:113984. doi: 10.1016/j.jep.2021.113984 33711438

[84] Nibouche-Hattab W-N. ÉTUDE de la MORBIDITÉ AU MOMENT DU DIAGNOSTIC DU DIABÈTE DE TYPE 2 DE L’ADULTE [Study of Morbidity at the Time of Adult Type 2 Diabetes Diagnosis]. Algiers University Benyoucef Benkhedda (2015).

[85] Sellam Y Malek R Semrouni M Belhadj M Zekri S Brouri M. PDB37 cost-effectiveness of insulin degludec/insulin aspart twice daily versus BASAL-bolus full regimen in patients with type 2 diabetes mellitus in Algeria. Value Health. (2020) 23:S512. doi: 10.1016/j.jval.2020.08.634

[86] Malek R Arbouche Z Belhadj M Nicolucci A Gari S. Corrélation entre l’hypoglycémie sévère et l’hypoglycémie non-sévère et les résultats psychologiques chez les personnes algériennes atteintes de diabète de type 1 et de type 2: résultats de l’étude DAWN2™. Méd Des. Maladies Métaboliques. (2017) 11:293–9. doi: 10.1016/S1957-2557(17)30070-6

[87] Ayad F Belhadj M Pariés J Attali JR Valensi P. Association between cardiac autonomic neuropathy and hypertension and its potential influence on diabetic complications. Diabetic Med. (2010) 27:804–11. doi: 10.1111/j.1464-5491.2010.03027.x 20636962

[88] Amrane M Houcher Z Begag S Houcher B Benlatreche C Touabti A. Influence of retinopathy on plasma concentrations of total homocysteine and other biochemical parameters in Algerian patients with type 2 diabetes mellitus. Pteridines. (2012) 23:96–103. doi: 10.1515/pteridines.2012.23.1.96

[89] Aouiche S Ouerdane K Frioui M Ait Boudaoud A Ragguem A Boudiba A. Neuropathie diabétique douloureuse: fréquence, facteurs de risque et gravité dans une cohorte de 400 sujets diabétiques en Algérie. Méd Des. Maladies Métaboliques. (2014) 8:211–5. doi: 10.1016/S1957-2557(14)70743-6

[90] Boukheloua M Berrehal M Baali A Chelghoum S Nibouche D. Prevalence and predictive factors of severe coronary lesions in Algerian patients undergoing coronary angiography. Open J. Pathol. (2023) 13:184–94. doi: 10.4236/ojpathology.2023.134019

[91] Maammar F Regagba D Attar S Snoussaoui Y Benamar C Belbachir F. P127 Profil épidémiologique d’une population des diabétiques à Tlemcen; Algérie. Diabetes Metab. (2014) 40:A59. doi: 10.1016/S1262-3636(14)72419-X

[92] Global Burden of Disease Collaborative Network, and Institute for Health Metrics and Evaluation (IHME). Global Burden of Disease Study 2019 (GBD 2019) Results (2020). Available online at: https://vizhub.healthdata.org/gbd-results/ (Accessed September 10, 2024).

[93] El-Kebbi IM Bidikian NH Hneiny L Nasrallah MP. Epidemiology of type 2 diabetes in the Middle East and North Africa: Challenges and call for action. World J. Diabetes. (2021) 12:1401–25. doi: 10.4239/wjd.v12.i9.1401 PMC8472500 34630897

[94] Kengne AP Bentham J Zhou B Peer N Matsha TE Bixby H. Trends in obesity and diabetes across Africa from 1980 to 2014: an analysis of pooled population-based studies. Int. J. Epidemiol. (2017) 46:1421–32. doi: 10.1093/ije/dyx078 PMC5837192 28582528

[95] Malek R. Prevalence of type 2 diabetes mellitus in Africa: an updated narrative review. North Afr. J. Food Nutr. Res. (2021) 4:S87–92. doi: 10.51745/najfnr.4.9.S87-S92

[96] Roche MM Wang PP. Sex differences in all-cause and cardiovascular mortality, hospitalization for individuals with and without diabetes, and patients with diabetes diagnosed early and late. Diabetes Care. (2013) 36:2582–90. doi: 10.2337/dc12-1272 PMC3747934 23564923

[97] Herman WH Ye W Griffin SJ Simmons RK Davies MJ Khunti K. Early detection and treatment of type 2 diabetes reduce cardiovascular morbidity and mortality: A simulation of the results of the anglo-danish-dutch study of intensive treatment in people with screen-detected diabetes in primary care (ADDITION-europe). Diabetes Care. (2015) 38:1449–55. doi: 10.2337/dc14-2459 PMC4512138 25986661

[98] ElSayed NA Aleppo G Aroda VR Bannuru RR Brown FM Bruemmer D. 3. Prevention or delay of diabetes and associated comorbidities: standards of care in diabetes—2023. Diabetes Care. (2023) 46:S41–8. doi: 10.2337/dc23-S003 PMC9810464 36507633

[99] Myerson R Lu T Peters A Fox S Huang E. Impact of health insurance policy on diabetes management. In: Behavioral Diabetes. Springer International Publishing, Cham (2020). p. 491–504. doi: 10.1007/978-3-030-33286-0_31

[100] Chauhan BF Jeyaraman M Mann AS Lys J Skidmore B Sibley KM. Behavior change interventions and policies influencing primary healthcare professionals’ practice—an overview of reviews. Implementation Sci. (2017) 12:3. doi: 10.1186/s13012-016-0538-8 PMC5216570 28057024

[101] ElSayed NA Aleppo G Aroda VR Bannuru RR Brown FM Bruemmer D. 1. Improving care and promoting health in populations: standards of care in diabetes—2023. Diabetes Care. (2023) 46:S10–8. doi: 10.2337/dc23-S001 PMC9810463 36507639

[102] Inzucchi SE Bergenstal RM Buse JB Diamant M Ferrannini E Nauck M. Management of hyperglycemia in type 2 diabetes: a patient-centered approach: position statement of the American Diabetes Association (ADA) and the European Association for the Study of Diabetes (EASD). Diabetes Care. (2012) 35:1364–79. doi: 10.2337/dc12-0413 PMC3357214 22517736

[103] Chan JCN Lim L-L Wareham NJ Shaw JE Orchard TJ Zhang P. The Lancet Commission on diabetes: using data to transform diabetes care and patient lives. Lancet. (2020) 396:2019–82. doi: 10.1016/S0140-6736(20)32374-6 33189186

[104] Williams DM Jones H Stephens JW. Personalized type 2 diabetes management: an update on recent advances and recommendations. Diabetes Metab. Syndr. Obes. (2022) 15:281–95. doi: 10.2147/DMSO.S331654 PMC8824792 35153495

